# How to Rejuvenate

**Published:** 1858-12

**Authors:** 


### HOW TO REJUVENATE.

A CORRESPONDENT in sight of the nineties writes: "My wife is about forty years younger than myself, the picture of health, handsome, intelligent, a liberal education, an excellent reader, always cheerful, and a good cook; hence, I have renewed my age since we became one."

That is to say, if any old codger of a bach or widower would like to feel young again, let him spruce up, and get a handsome, handy, cheerful, and good-natured young wife—the "handy" and the good-nature especially.

